# Pharmacy-Led Digital Education for Rational Antibiotic Use in Companion Animals: A Randomized Controlled Trial

**DOI:** 10.3390/antibiotics15060592

**Published:** 2026-06-09

**Authors:** Chanuttha Ploylearmsang, Chayanan Panmawong, Thanwarat Chinnachak, Jaroon Wandee

**Affiliations:** 1 Social Pharmacy Research Unit, Faculty of Pharmacy, Mahasarakham University, Maha Sarakham 44150, Thailand; 2 Faculty of Pharmacy, Mahasarakham University, Maha Sarakham 44150, Thailand; 63010710019@msu.ac.th (C.P.); 63010710036@msu.ac.th (T.C.); 3 Faculty of Veterinary Sciences, Mahasarakham University, Maha Sarakham 44000, Thailand; jaroon.w@msu.ac.th

**Keywords:** companion animals, antibiotic use, antimicrobial resistance, pharmacy-led education, digital health education, undergraduate students, one health

## Abstract

**Background:** Inappropriate antibiotic use in companion animals contributes to antimicrobial resistance within the One Health context. Educational interventions targeting non-health companion animal owners, particularly undergraduate students who frequently make day-to-day animal care decisions, have remained limited. **Methods:** A randomized controlled trial was conducted among undergraduate students who owned companion animals in Maha Sarakham Province, Thailand. Participants were randomly assigned to a research group (n = 31) or a control group (n = 33). The research group received pharmacy-led digital educational materials, including videos and posters, delivered via an online platform over three consecutive days, while the control group received no educational materials. Antibiotic knowledge was assessed using a structured questionnaire before and after the intervention in both groups. Confidence in rational antibiotic use and satisfaction with the educational materials were assessed in the research group. Nonparametric statistical tests were applied for within- and between-group comparisons. **Results:** Baseline characteristics and antibiotic knowledge scores were comparable between groups. After the intervention, the research group demonstrated significantly higher antibiotic knowledge scores than the control group (9.58 ± 0.76 vs. 7.82 ± 1.31; *p* < 0.001). The mean improvement in knowledge score was greater in the research group (Δ = 1.55 vs. 0.09; *p* < 0.001). Confidence in rational antibiotic use increased significantly following the intervention (*p* < 0.001). **Conclusions:** Pharmacy-led digital education improved antibiotic knowledge and confidence among undergraduate companion animal owners, supporting the role of pharmacists in antimicrobial stewardship within a One Health framework.

## 1. Introduction

The ownership of companion animals, particularly dogs and cats, has increased globally across all age groups, including university students [1]. Companion animals are increasingly regarded as family members and sources of emotional support, contributing positively to owners’ mental [2] and social well-being [3]. However, this close human–animal relationship also raises important public health concerns, particularly regarding the use of antibiotics in companion animals and its implications for antimicrobial resistance (AMR) [4].

Antimicrobial resistance (AMR) has been recognized as one of the most pressing global public health threats, prompting coordinated international policy responses under the One Health framework [5]. The World Health Organization (WHO), the Food and Agriculture Organization of the United Nations (FAO), and the World Organisation for Animal Health (WOAH) have jointly emphasized that inappropriate antimicrobial use in humans and animals, including companion animals, plays a critical role in accelerating the emergence and spread of resistant pathogens. The Global Action Plan on Antimicrobial Resistance calls for multisectoral strategies that extend beyond clinical prescribing practices to address antimicrobial use behaviors among the general public and animal owners. The use of antibiotics in companion animals has been increasingly linked to the emergence and transmission of resistant bacteria, posing a direct threat to animal health, treatment outcomes, and survival, while also facilitating the spread of resistant pathogens between animals and humans through direct contact or shared environments [6]. Evidence has demonstrated that antimicrobial-resistant bacteria such as *Staphylococcus* spp. and resistant *Enterobacteriaceae* can be shared between companion animals and their owners, highlighting the zoonotic risks associated with inappropriate antibiotic use in household pets within a One Health context.

In veterinary practice, antibiotics are often prescribed to treat bacterial infections, sometimes empirically when diagnostic testing is unavailable or delayed [7]. Inappropriate antibiotic use, such as incorrect dosing, premature discontinuation, or use without veterinary consultation can accelerate the development of antimicrobial resistance, undermining treatment effectiveness in both animals and humans [8].

International policy documents from WHO, FAO, and WOAH explicitly highlight education, awareness, and responsible antimicrobial use at the community level as key pillars of antimicrobial stewardship, particularly in low- and middle-income countries (LMICs), where regulatory enforcement and access to professional veterinary care may be variable. Previous studies conducted in various countries have shown that companion animal owners often demonstrate limited knowledge and misconceptions regarding antibiotic use and AMR [9]. Although many owners acknowledge antimicrobial resistance as a serious problem, fewer understand that antibiotic use in animals can contribute to resistance affecting human health. Antimicrobial resistance in companion animals has also been reported in Thailand [10].

Common misconceptions include the belief that antibiotics are effective against viral infections, that resistance develops within the animal’s body rather than in bacteria, or that antibiotics can be used preventively. Similar knowledge gaps have been reported among university students, even among those with moderate general awareness of antibiotics, highlighting the need for targeted educational interventions [11,12].

Despite growing recognition of the importance of antimicrobial stewardship in veterinary settings, most educational interventions to date have primarily targeted veterinarians or pet owners in clinical contexts [13,14,15]. There is comparatively limited evidence on interventions aimed at non-health undergraduate students who own companion animals, a group that frequently makes day-to-day decisions regarding animal care and medication use. University students increasingly rely on digital sources, particularly social media, for health-related information, which may expose them to inaccurate or misleading content about antibiotic use [16].

Pharmacists are well-positioned to contribute to antimicrobial stewardship beyond human healthcare through education and public engagement. Pharmacy-led educational interventions have demonstrated effectiveness in improving antibiotic knowledge and promoting rational drug use in human health contexts [17]. However, evidence regarding the role of pharmacists in educating companion animal owners particularly through digital platforms remains scarce, especially in low- and middle-income countries.

Digital health education, including the use of videos and visual media, has gained increasing attention as an accessible and scalable approach to improving health literacy [18]. Electronic media may be particularly effective for younger populations by enhancing engagement, comprehension, and knowledge retention [19]. Nevertheless, randomized controlled trials evaluating the impact of pharmacy-led digital education on antibiotic knowledge and confidence related to companion animal care are limited.

Therefore, the aim of this study was to evaluate the effectiveness of pharmacy-led digital educational interventions, delivered through videos and posters, on antibiotic knowledge and confidence regarding rational antibiotic use in companion animals among undergraduate students in Maha Sarakham Province, Thailand. Such interventions align with global recommendations emphasizing task-sharing, interdisciplinary collaboration, and the use of digital health technologies to expand the reach of antimicrobial stewardship efforts, particularly in resource-limited settings. It was hypothesized that students who received the digital educational intervention would demonstrate significantly greater improvements in antibiotic knowledge and confidence compared with those who did not receive the intervention.

## 2. Results

### 2.1. Participant Baseline Characteristics

A total of 66 undergraduate students met the eligibility criteria and were randomized in a 1:1 ratio to the control group (n = 33) or the research group (n = 33). During follow-up, two participants in the research group lost contact, resulting in a final analytical sample of 33 participants in the control group and 31 participants in the research group.

Baseline demographic and background characteristics are presented in Table 1. Participants in both groups were predominantly female and first-year undergraduate students. Cats and dogs were the most owned companion animals. No statistically significant differences were observed between groups for demographic variables, academic characteristics, companion animal ownership, caregiving experience, or monthly income (*p* > 0.05 for all comparisons), indicating comparable baseline characteristics.

A statistically significant difference was observed in one baseline variable: participants in the control group reported obtaining antibiotic-related information from X (formerly Twitter) more frequently than those in the research group (*p* = 0.011).

### 2.2. Baseline Antibiotic Use Practices

Baseline practices related to antibiotic use in companion animals are summarized in Table 2. At pre-intervention, no statistically significant differences were identified between the control and research groups across all six practice-related items (*p* > 0.05).

At baseline, both groups demonstrated similar behavioral patterns. Appropriate practices were commonly reported for consulting veterinarians prior to antibiotic use and avoiding doubling after missed administration. Suboptimal practices were also observed in both groups, particularly regarding the management of missed doses and early discontinuation of antibiotics when animals appeared clinically improved.

### 2.3. Antibiotic Knowledge Outcomes

#### 2.3.1. Item-Level Knowledge Responses

Item-level comparisons of antibiotic knowledge before and after the intervention are presented in Table 3. At baseline, the proportions of correct responses did not differ significantly between the control and research groups for any of the knowledge items (*p* > 0.05). Within the control group, no significant changes in correct responses were observed between pre-test and post-test assessments. In contrast, the research group showed significant improvements in selected knowledge items following exposure to the educational materials. Post-intervention comparisons between groups demonstrated significantly higher correct response rates in the research group for items related to (*p* < 0.05 for all comparisons):

- differentiation between antibiotics, disinfectants, and anti-inflammatory drugs;
- recognition that antibiotics are not effective against viral infections;
- understanding the importance of completing the full prescribed antibiotic course;
- awareness that antibiotics should not be used preventively due to changes in weather conditions.

#### 2.3.2. Overall Knowledge Scores (Primary Outcome)

Overall antibiotic knowledge scores before and after the intervention are summarized in Table 4. At baseline, no significant difference in mean knowledge scores was identified between the control and research groups (7.91 ± 1.76 vs. 8.03 ± 1.66; *p* = 0.700).

Within-group analysis showed no significant change in the control group between pre-test and post-test scores (7.91 ± 1.76 vs. 7.82 ± 1.31; *p* = 0.518). In contrast, the research group showed a statistically significant increase in mean knowledge scores following the intervention (8.03 ± 1.66 vs. 9.58 ± 0.76; *p* < 0.001).

Between-group analysis at post-test revealed significantly higher knowledge scores in the research group compared with the control group (9.58 ± 0.76 vs. 7.82 ± 1.31; *p* < 0.001). The mean change in knowledge score was 1.55 points in the research group and 0.09 points in the control group, resulting in a statistically significant between-group difference (*p* < 0.001).

### 2.4. Confidence in Antibiotic Use (Secondary Outcome)

Changes in confidence regarding rational antibiotic use in companion animals among participants in the research group are presented in Table 5. Mean confidence scores increased significantly following the intervention, from 3.67 ± 0.96 at baseline to 4.92 ± 0.28 post-intervention (*p* < 0.001).

### 2.5. Satisfaction with Digital Educational Materials

Satisfaction with pharmacy-led digital educational materials among participants in the research group is shown in Table 6. Overall satisfaction levels were high for both poster-based and video-based materials.

For poster-based materials, the highest mean satisfaction score was reported for the clarity of illustrations (4.99 ± 0.20). For video-based materials, participants reported high levels of satisfaction for content structure and illustrative clarity (4.92 ± 0.28). High satisfaction ratings were consistently observed across all evaluated domains.

## 3. Discussion

This randomized controlled trial demonstrates that pharmacy-led digital education delivered through videos and posters significantly improved both antibiotic knowledge and confidence regarding rational antibiotic use in companion animals among undergraduate students. By focusing on companion animal owners rather than healthcare professionals, this intervention addresses a critical yet often overlooked behavioral determinant of antimicrobial use at the human–animal interface, which is central to the One Health approach [4,5,6].

The significant improvement in antibiotic knowledge observed in the research group is consistent with previous studies showing that targeted educational interventions can effectively correct misconceptions about antibiotic use and antimicrobial resistance among non-professional populations [11,15,20]. In particular, the largest gains were observed in knowledge items related to antibiotic ineffectiveness against viral infections, the necessity of completing prescribed courses, and the inappropriateness of preventive antibiotic use, which have been repeatedly identified as persistent misconceptions among companion animal owners and university students [8,9,11].

These findings align with randomized trials conducted in other settings, where short, focused digital interventions, such as animated videos or concise educational materials, were shown to be particularly effective in correcting misinformation rather than merely reinforcing existing knowledge [15,20]. The lack of significant improvement in some knowledge items may reflect relatively high baseline knowledge levels among participants, a phenomenon also reported in previous studies involving educated young adults [11,12]. Alternatively, it may indicate that certain concepts, such as mechanisms of antimicrobial resistance, require more in-depth or repeated educational exposure.

Importantly, the observed improvements were consistent with national veterinary rational drug use guidelines in Thailand, which emphasize appropriate indications, adherence to prescribed duration, and avoidance of unnecessary antibiotic use in animals [21]. However, antimicrobial stewardship in companion animal practice is challenged by limitations in existing antimicrobial use guidelines. In veterinary medicine, species-specific interpretative criteria for antimicrobial susceptibility testing remain limited, and guidelines such as those issued by the Clinical and Laboratory Standards Institute (CLSI) do not cover all companion animal species or commonly used veterinary antibiotics. In addition, guidance for antibiotics intended exclusively for veterinary use is often less comprehensive than that available for human medicines. These gaps may contribute to empirical prescribing and owner misunderstandings, further underscoring the importance of clear, accessible educational interventions for companion animal owners.

This suggests that pharmacy-led educational content can effectively translate professional guidelines into accessible messages for lay companion animal owners. This model is highly transferable to other low- and middle-income country (LMIC) settings, where pharmacists are accessible healthcare professionals and digital communication platforms are widely used. The low resource requirements, interdisciplinary design, and adaptability of the intervention support its potential scalability and integration into broader One Health-oriented antimicrobial stewardship programs. Beyond knowledge acquisition, the intervention resulted in a marked increase in participants’ confidence regarding rational antibiotic use for companion animals. Confidence is a critical psychosocial determinant of behavior, influencing individuals’ willingness to seek professional advice, adhere to recommendations, and resist misinformation [22]. Prior research has shown that low confidence among non-health professionals is associated with self-directed antibiotic use and reliance on informal information sources, including social media [16,22].

The substantial increase in confidence observed in this study suggests that pharmacy-led education may empower undergraduate companion animal owners to engage more effectively with veterinarians, ask informed questions, and adhere to veterinary prescriptions. This is particularly relevant in contexts where companion animal owners may have easy access to antibiotics or receive advice from non-professional sources, thereby increasing the risk of inappropriate use [8,9].

### 3.1. Role of Digital Media and Pharmacy-Led Education

The effectiveness of the intervention is likely attributable to the combined impact of digital delivery and professional leadership by pharmacists. Digital media formats, particularly videos and visually engaging posters, have been shown to enhance comprehension, engagement, and knowledge retention among younger populations accustomed to online learning environments [18,19]. The high satisfaction scores reported for both formats in this study further support the acceptability and feasibility of digital education as a scalable intervention.

Pharmacists are increasingly recognized as key contributors to antimicrobial stewardship across healthcare systems, particularly through patient education and public engagement [17]. While their role in veterinary antimicrobial stewardship has been less explored, the present findings suggest that pharmacists can effectively extend their educational role beyond human medicine. Pharmacy-led education may complement veterinary services by reinforcing consistent messages about antibiotic use, adherence, and resistance, thereby supporting a multidisciplinary One Health approach [4,5,21].

### 3.2. Implications for Antimicrobial Stewardship and One Health

The findings of this study have important implications for antimicrobial stewardship in companion animal care. In Thailand, antimicrobial resistance and rational drug use are not systematically addressed in primary or secondary general education curricula. Formal education on antimicrobial stewardship is typically introduced at the tertiary level, particularly within health science and veterinary programs. While veterinary curricula include antimicrobial use and resistance, companion animal owners without health-related training may have limited exposure to these concepts. This educational gap highlights the value of supplementary interventions, such as pharmacy-led digital education, to raise awareness and promote responsible antibiotic use among non-health undergraduate students who care for companion animals [18]. Undergraduate students represent a substantial and influential group of companion animal owners who frequently make day-to-day decisions regarding animal health and medication use. Improving their knowledge and confidence through structured, pharmacy-led education may contribute to more responsible antibiotic use and reduce behaviors that drive antimicrobial resistance [6,9,10].

From a One Health perspective, interventions targeting non-professional animal owners are essential, as antimicrobial resistance is shaped not only by prescribing practices but also by user behavior across human and animal populations [4,5,6]. Integrating pharmacy-led digital education into university health programs, veterinary outreach initiatives, or community-based antimicrobial stewardship strategies could represent a cost-effective and scalable approach to addressing antimicrobial resistance at the human–animal interface.

### 3.3. Strengths and Limitations

This study has several strengths. The randomized controlled design enhances internal validity, and the intervention was grounded in interdisciplinary expertise from both pharmacy and veterinary disciplines. The use of digital media increases the potential for scalability and real-world implementation, particularly in resource-limited settings.

However, several limitations should be acknowledged. First, the short intervention period and brief follow-up limited our ability to assess long-term knowledge retention, sustained confidence, or enduring behavioral change related to antibiotic use in companion animals.

Second, outcomes were based on self-reported measures, which may be subject to social desirability or response bias. Third, the study was conducted in a single province and primarily involved undergraduate students, limiting generalizability to other populations of companion animal owners. Fourth, simple randomization without stratification was used. While baseline characteristics were largely comparable between groups, this method may introduce imbalances in relatively small samples. Although only one baseline variable differed significantly between groups, future studies should consider stratified or block randomization strategies to further enhance group comparability. In addition, although knowledge and confidence are important precursors to behavior change, actual antibiotic use behaviors were not objectively assessed, which limits the ability to draw conclusions regarding real-world practice change.

### 3.4. Future Research

Future studies should incorporate longer follow-up periods and repeated assessments to evaluate the durability of knowledge gains, confidence, and antibiotic use behaviors over time. Expanding the intervention to diverse populations, including older companion animal owners and different cultural contexts, would enhance generalizability. Comparative studies evaluating different digital formats or interdisciplinary delivery models may also provide insights into optimizing educational effectiveness and antimicrobial stewardship outcomes.

## 4. Research Methods

### 4.1. Study Design

This study employed a randomized controlled trial (RCT) design to evaluate the effectiveness of a pharmacy-led digital educational intervention on antibiotic knowledge and confidence regarding rational antibiotic use in companion animals. The study was conducted between November 2024 and January 2025 in Maha Sarakham Province, Thailand. The trial was retrospectively registered with the Thai Clinical Trials Registry (TCTR20260601013) on 1 June 2026 (https://www.thaiclinicaltrials.org/show/TCTR20260601013).

Ethical approval was obtained from the Human Research Ethics Committee of Mahasarakham University on 31 October 2024 (Approval No. 676–557/2024). All participants provided electronic informed consent prior to study participation.

### 4.2. Study Population and Eligibility Criteria

The study population consisted of undergraduate students enrolled during the 2024 academic year who owned at least one companion animal, including dogs, cats, small mammals (e.g., rodents and rabbits), or birds, and resided in or near the Mahasarakham University area.

Inclusion criteria were:

1. Undergraduate students aged 18 years or older;
2. Ownership of at least one companion animal;
3. Prior experience administering medications presumed to be antibiotics to their companion animals;
4. Ability to communicate and complete online questionnaires;
5. Access to an electronic device with internet connectivity;
6. Willingness to provide informed consent.

Exclusion criteria included:

- Withdrawal from the study during the intervention period;
- Inability to complete post-intervention assessment after at least three contact attempts via the LINE application.

### 4.3. Sample Size Calculation

The sample size was calculated using a standard formula for clinical research:

n = (zα + zβ)2σ^2^/(μ1 − μ0)^2^

where

zα = 1.96 (for 95% confidence);

zβ = 0.84 (for 80% power);

*σ* = 8.54 (based on Yotarlai, 2016) [23].

The standard deviation (SD = 8.54) was derived from a previously published study using a broader knowledge scale and was applied conservatively to ensure adequate statistical power.

*μ*1 − *μ*0 = 4.00 (expected mean difference).

Substituting these values yielded a sample size of approximately 28.15 per group. The minimum required sample size was calculated as 28 participants per group. To account for potential attrition and incomplete responses, an additional 10% was added. Consequently, 33 participants were allocated to each group, resulting in a total sample size of 66 participants.

### 4.4. Randomization and Allocation

Participants were randomly assigned in a 1:1 ratio to either the research group or the control group using a computer-generated random number sequence. Simple randomization was employed without stratification. Allocation was conducted by a researcher not involved in outcome assessment to minimize allocation bias. Due to the nature of the educational intervention, blinding of participants was not feasible. However, data analysis was performed using anonymized datasets.

### 4.5. Intervention

The intervention consisted of pharmacy-led digital educational materials, including short educational videos and visual posters focusing on appropriate antibiotic use in companion animals. The content was developed by two PharmD students under the supervision of a licensed pharmacist and a veterinarian.

The educational materials covered:

- Basic concepts of antibiotics;
- Differences between antibiotics, antivirals, anti-inflammatory agents, and disinfectants;
- Appropriate indications for antibiotic use in companion animals;
- Risks of inappropriate antibiotic use and antimicrobial resistance;
- Proper dosing, duration, and adherence to veterinary prescriptions.

Content validity and clarity were reviewed by one pharmacist and one veterinarian prior to implementation.

Participants allocated to the research group received pharmacy-led digital educational materials, including short videos and visually engaging posters focusing on rational antibiotic use in companion animals. The materials were delivered via the LINE application over three consecutive days, and participants were able to ask questions, which were answered by a supervising pharmacist.

Participants assigned to the control group did not receive any educational materials during the study period and continued to obtain information from their usual sources. Post-intervention assessments were administered to both groups three days after baseline data collection.

### 4.6. Data Collection and Research Instruments

The primary outcome of this study was antibiotic knowledge score, while secondary outcomes included confidence in antibiotic use and satisfaction with digital educational materials. Data was collected using a structured online questionnaire comprising four sections:

**Section 1**: Participant Characteristics

A 10-item questionnaire collected demographic and background information, including sex, field and year of study, type of companion animal, experience in animal care, sources of antibiotic-related information, and monthly income.

**Section 2**: Antibiotic Knowledge

Antibiotic knowledge was assessed using a 10-item multiple-choice questionnaire, adapted from previously published studies. Each correct response received one point, with total scores ranging from 0 to 10.

Knowledge levels were categorized as:

- High knowledge: 66.68–100%;
- Moderate knowledge: 33.34–66.67%;
- Low knowledge: 0–33.33%.

**Section 3**: Antibiotic Use Practices

Practices related to antibiotic use in companion animals were measured using a 6-item questionnaire with three response options: *Never*, *Sometimes*, and *Regularly*.

**Section 4**: Confidence and Satisfaction

Participants in the research group completed:

- A confidence assessment using a 5-point Likert scale (1 = very low to 5 = very high), evaluating confidence in rational antibiotic use for companion animals;
- A satisfaction questionnaire evaluating perceptions of digital educational materials.

### 4.7. Reliability and Validity

Questionnaire items were adapted from previously published studies on antibiotic use and antimicrobial resistance among students and companion animal owners. Content validity, clarity, and relevance were reviewed by a pharmacist and a veterinarian. Prior to data collection, the questionnaire was pilot-tested for clarity and comprehension among a small group of undergraduate students. Internal consistency reliability was assessed using Cronbach’s alpha, which demonstrated acceptable reliability for the knowledge, confidence, and satisfaction scales (Cronbach’s alpha ≥ 0.70).

### 4.8. Statistical Analysis

Descriptive statistics were used to summarize participant characteristics and outcome measures. Continuous variables were reported as means and standard deviations, while categorical variables were presented as frequencies and percentages. Normality of continuous data was assessed using the Shapiro–Wilk test. As outcome variables were not normally distributed, nonparametric statistical tests were applied. The Wilcoxon signed-rank test was used to compare pre- and post-intervention scores within groups. The Mann–Whitney U test was used to compare differences between groups. Categorical variables were analyzed using Pearson’s Chi-square test, Fisher’s Exact test, or McNemar’s test as appropriate.

All analyses were conducted using SPSS Statistics version 29.0. Statistical significance was set at *p* < 0.05.

## 5. Conclusions

This study demonstrates that pharmacy-led digital education can improve antibiotic knowledge and confidence regarding rational antibiotic use in companion animals among undergraduate students. While the findings support the potential role of pharmacists in antimicrobial stewardship at the community level, generalizability is limited to similar educational and demographic contexts. Broader scalability should be evaluated through larger, multi-site studies with longer follow-up and behavioral outcome assessments.

## Figures and Tables

**Table 1:** characteristics of Participants in the Control and Research groups.

Characteristic	Control Group n = 33 Number (%)	Research Group n = 31 Number (%)	*p* Value ^a^
**Sex**			0.414
Male	12 (36.4)	8 (25.8)	
Female	21 (63.6)	22 (71.0)	
LGBTQ+	0 (0.0)	1 (3.2)	
**Study Faculty**			0.939
Health sciences	5 (15.2)	5 (16.2)	
Science and technology	11 (33.4)	13 (38.8)	
Humanities and social sciences	17 (51.4)	13 (45.1)	
**Year of Study**			0.796
Year 1	10 (30.3)	9 (29.0)	
Year 2	9 (27.3)	6 (19.4)	
Year 3	8 (24.2)	8 (25.8)	
Year 4	6 (18.2)	7 (22.6)	
Other Year	0 (0.0)	1 (3.2)	
**Types of Companion Animal Owned**			0.192
Cat	15 (45.5)	15 (48.4)	
Dog	14 (42.4)	11 (35.5)	
Rabbit	4 (12.1)	1 (3.2)	
Hamster	0 (0.0)	2 (6.5)	
Bird	0 (0.0)	2 (6.5)	
**Companion Animal Acquisition Method**			0.386
Rescued/Collected	13 (39.4)	8 (25.8)	
Purchased	9 (27.3)	13 (41.9)	
Gifted by Others	11 (33.3)	10 (32.3)	
**Experience in Raising Companion Animal**			0.445 ^b^
No experience	7 (21.2)	8 (25.8)	
Experienced	26 (78.8)	23 (74.2)	
**Surrounding situation of Animal Ownership**			0.590
No peers or acquaintances who own companion animal	2 (6.1)	3 (9.7)	
There are peers or acquaintances who own companion animal	31 (93.9)	28 (90.3)	
**Companion Animal Care Responsibility**			0.332 ^b^
Raising alone	6 (18.2)	8 (25.8)	
Raising with others	27 (81.8)	23 (74.2)	
**Monthly Income (Baht)**			0.888
Less than 2000	0 (0.0)	1 (3.2)	
2001–5000	8 (24.2)	7 (22.6)	
5001–8000	16 (48.5)	14 (45.2)	
8001–10,000	4 (12.1)	4 (12.90	
More than 10,000	5 (15.2)	5 (16.1)	
**Sources of Information about Antibiotics**			
Peers or Acquaintances	12 (36.4)	18 (58.1)	0.068 ^b^
Television	8 (24.2)	8 (25.8)	0.557 ^b^
TikTok	28 (84.8)	21 (67.7)	0.093 ^b^
Facebook	16 (48.5)	16 (51.6)	0.500 ^b^
Line	8 (24.2)	5 (16.1)	0.311 ^b^
X (Twitter)	26 (78.8)	15 (48.4)	0.011 ^b^*
Brochure/Flyers	6 (18.2)	6 (19.4)	0.578 ^b^

Note: ^a^ Pearson Chi-square test, ^b^ Fisher’s Exact Test, * Significance at α = 0.05.

**Table 2:** Comparison of Correct Antibiotic Use Practices Before Receiving Knowledge Between the Control and Research groups.

Question	Level of Practice	Number of Respondents in the Control Group n = 33 (%)	Number of Respondents in the Research Group n = 31 (%)	*p*-Value ^a^ (Between Groups)
1. Following antibiotic use in companion animals as advised by knowledgeable individuals such as veterinarians	Never Sometimes Regularly	4 (12.1)	5 (16.1)	0.788
		12 (36.4)	9 (29.0)	
		17 (51.5)	17 (54.80)	
2. Consulting a veterinarian before administering antibiotics to companion animals	Never Sometimes Regularly	2 (6.1)	5 (16.1)	0.400
		8 (24.2)	8 (25.8)	
		23 (69.7)	18 (58.1)	
3. Stopping antibiotics immediately when the companion animal’s clinical symptoms improved, as they are no longer necessary	Never Sometimes Regularly	17 (51.5)	16 (51.6)	0.062
		9 (27.3)	14 (45.2)	
		7 (21.2)	1 (3.2)	
4. Using antibiotics for all companion animals according to veterinary prescriptions	Never Sometimes Regularly	3 (9.1)	5 (16.1)	0.581
		5 (15.2)	6 (19.4)	
		25 (75.8)	20 (64.5)	
5. Administering a missed antibiotic dose as soon as it is remembered	Never Sometimes Regularly	9 (27.3)	9 (29)	0.778
		14 (42.4)	15 (48.4)	
		10 (30.3)	7 (22.6)	
6. Doubling the next antibiotic dose when a companion animal’s medication is forgotten	Never Sometimes Regularly	28 (84.8)	29 (93.5)	0.228
		2 (6.1)	2 (6.5)	
		3 (9.1)	0 (0.0)	

Note: ^a^ Pearson Chi-square test.

**Table 3:** Number of Correct Responses About Antibiotic Knowledge Before and After Receiving Knowledge in the Control and Research groups.

Question	Control Group Correct Responses (%) (n = 33)		Research Group Correct Responses (%) (n = 31)		*p*-Value ^a^ (Pre-Test Between Groups)	*p*-Value ^a^ (Post-Test Between Groups)
	Pre-Test	Post- Test	Pre- Test	Post-Test		
1. Antibiotics, disinfectants, and anti-inflammatory drugs are the same	22 (66.7)	21 (63.6)	27 (87.1)	31 (100.0)	0.050 ^b^	<0.001 *
***p*-value ^c^**	1.000		0.134			
2. Antibiotics are classified as dangerous drugs under the Drug Act of 1967 (B.E. 2510)	30 (90.9)	25 (75.8)	23 (74.2)	29 (93.5)	0.074 ^b^	0.051 ^b^
***p*-value ^c^**	0.074		0.077			
3. Antibiotics can kill viruses	14 (42.4)	14 (42.4)	18 (58.1)	28 (90.3)	0.159 ^b^	<0.001 ^b^*
***p*-value ^c^**	1.000		0.004 *			
4. Antibiotic dosage must follow the drug label instructions	31 (93.9)	31 (93.9)	29 (93.5)	30 (96.8)	0.949	0.592
***p*-value ^c^**	1.000		1.000			
5. The use of antibiotics in companion animals should always be consulted with a veterinarian	33 (100.0)	33 (100.0)	29 (93.5)	31 (100.0)	0.138	n/a
***p*-value ^c^**	n/a		0.480			
6. Antibiotics should only be used to treat sick animals	30 (90.9)	32 (97.0)	29 (93.5)	28 (90.3)	0.694	0.272
***p*-value ^c^**	0.480		1.000			
7. Antibiotics can be stopped when the companion animal when clinical symptoms improved, even if the full duration prescribed on the drug label or by the veterinarian has not been completed	23 (69.70	22 (66.7)	24 (77.4)	29 (93.5)	0.340 ^b^	0.008 ^b^*
***p*-value ^c^**	1.000		0.074			
8. Long-term use of traditional antibiotics in companion animals does not cause drug resistance	21 (63.6)	22 (66.7)	24 (77.4)	24 (77.4)	0.176 ^b^	0.249 ^b^
***p*-value ^c^**	1.000		1.000			
9. Antibiotics should be given preventively when weather changes may affect animal health	25 (75.8)	25 (75.8)	25 (80.6)	31 (100.0)	0.433 ^b^	0.003 *
***p*-value ^c^**	1.000		0.041 *			
10. The unnecessary use of antibiotics in companion animals may lead to drug resistance	32 (97.0)	33 (100.0)	29 (93.5)	31 (100.0)	0.518	n/a
***p*-value ^c^**	1.000		0.480			

Note: ^a^ Pearson Chi-square test ^b^ Fisher’s Exact Test ^c^ McNemar Chi-square test * Significance at α = 0.05.

**Table 4:** Comparison of Knowledge Scores Before and After Receiving Knowledge About Antibiotics Within and Between the Control and Research groups.

Sample	Average Score (Out of 10) (Mean ± SD)		(Δ) Score Difference	Percentage of Improvement	*p*-Value ^d^ (Within Group)
	Pre-Test	Post-Test			
**Control Group (n = 33)**	7.91 ± 1.76	7.82 ± 1.31	0.09	High (78.2)	0.518
**Research group (n = 31)**	8.03 ± 1.66	9.58 ± 0.76	1.55	High (95.8)	<0.001 *
***p*-value ^e^** (Between groups)	0.700	<0.001 *	<0.001 *		

Note: ^d^ Wilcoxon Signed Rank Test, ^e^ Mann–Whitney U Test, * Significance at α = 0.05.

**Table 5:** Comparison of Confidence in Using Antibiotics in Companion Animals Before and After Receiving Knowledge in the Research group.

Confidence in Using Antibiotics in Companion Animals	Mean ± SD Score Before Intervention (Out of 5)	Mean ± SD Score After Intervention (Out of 5)	*p*-Value ^d^ (Pre-Test 2 Groups)
**Research group** **(n = 31)**	3.67 ± 0.96	4.92 ± 0.28	<0.001 *

Note: ^d^ Wilcoxon Signed Rank Test, * Significance at α = 0.05.

**Table 6:** Satisfaction Scores for Receiving Knowledge Through Electronic Media in the Research group.

Satisfaction Items	Frequency (%) n = 24		Mean ± SD (Out of 5)	Interpretation
	High Satisfied	Highest Satisfied		
**Poster Format**				
(1) Content is easy to read	4 (16.7)	20 (83.3)	4.83 ± 0.38	High
(2) Content is interesting	3 (12.5)	21 (87.5)	4.88 ± 0.34	High
(3) Design is visually appealing	4 (16.7)	20 (83.3)	4.83 ± 0.38	High
(4) Illustrations enhance understanding	1 (4.2)	23 (95.8)	4.99 ± 0.20	High
**Video Format**				
(1) The video has well-structured content	2 (8.3)	22 (91.7)	4.92 ± 0.28	High
(2) content is simple and easy to understand	6 (25.0)	18 (75.0)	4.75 ± 0.44	High
(3) The content is engaging	4 (16.7)	20 (83.3)	4.83 ± 0.38	High
(4) Illustrations enhance understanding	2 (8.3)	22 (91.7)	4.92 ± 0.28	High

Note: Only 24 participants in the research group completed all satisfaction questionnaire items; therefore, satisfaction analysis is based on this subset of respondents.

## Data Availability

The datasets generated during and/or analyzed during the current study are available from the corresponding author on reasonable request.
